# Dental management of scleroderma patients using pentoxifylline plus vitamin E with and without TheraBite^®^ to reduce trismus: Two case reports and brief review of literature

**DOI:** 10.1002/ccr3.2572

**Published:** 2020-01-17

**Authors:** Daniel N. Reed, David L. Hall, James H. Cottle, Katherine Frimenko, Christina K. Horton, Farah Abu Sharkh, Rachel Beckett, Brandon Hernandez, Hannah Mabe, Shadee T. Mansour, Sebastian A. Rodriguez, Bradley Weprin, Leigh E. Yarborough

**Affiliations:** ^1^ GPR Ohio State University College of Dentistry Columbus OH USA

**Keywords:** pentoxifylline, scleroderma, Thera Bite®, trismus, VitE

## Abstract

To provide evidence supporting the off label use of pentoxifylline and vitamin E especially by dentists with TheraByte to reduce trismus in scleroderma patients.

## Graphical abstract

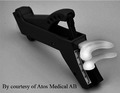

## 1 INTRODUCTION

To successfully manage dental patients with scleroderma. Two patients diagnosed with scleroderma presented to the Ohio State University General Practice Residency clinic for routine dental care. Both patients exhibited significantly reduced opening of their jaws. One patient was prescribed pentoxifylline 800‐1200 mg. plus vitamin E 800 IU daily. The second patient received pentoxifylline plus vitamin E and performed Thera Bite® jaw exercises four times daily. Both patients jaw openings improved markedly. Addition of TheraBite® increased the effect of pentoxifylline plus vitamin E. No side effects or complications were observed. Pentoxifylline, Vitamin E and Thera Byte® help reduce trismus in scleroderma patients.

Scleroderma is an incurable chronic multisystem autoimmune connective tissue disorder of unknown etiology.^1^, ^2^ Scleroderma is divided into localized scleroderma and systemic sclerosis.^2^

Localized scleroderma is thickening, hardening, and vasculopathy of skin.^1^, ^2^ Localized scleroderma is caused by excess collagen production and intense fibrosis in skin sometimes involving underlying muscle and bone.^2^ The prevalence of localized scleroderma in the United States is 1/500‐1/900,^3^ with an incidence rate of 27 new cases per million population per year.^2^, ^3^

Systemic sclerosis (SSc) usually begins as Raynaud's phenomenon^4^ with blanching and cyanosis of digits due to vasospasm of diseased blood vessels in response to cold or emotional stress.^5^ Raynaud's may last for many years in limited scleroderma, or may have a short duration before more symptoms develop.^1^ The initial edematous phase consists of arthralgia and swelling of the hands and feet. The indurative phase involves internal organs. After 3‐4 years of fibrosis, disease progression appears to stabilize,^2^ and patients often experience an improvement in skin thickening.^6^ However, organ system damage can continue to cause debilitating problems. Rapid tightening (sclerodactyly) often leads to severe flexion contractures with claw‐like hand deformities. Patients may have a firm mask‐like facial appearance, with a pinched nose from nasal alar atrophy. Cutaneous involvement occurs first in 80% of all patients within 3 years of diagnosis.^7^ Systemic effects can involve the esophagus, heart, and lungs. Serious muscular, gastrointestinal, pulmonary, renal, and cardiac conditions also result from internal organ fibrosis and muscular atrophy. Scleroderma pulmonary disease is the most frequent cause of death.^8^

SSc occurs in approximately 1/4000 of population in the United States, far higher than in other countries,^9^, ^10^ with 19 new cases per million per year.^11^, ^12^ There is a female preponderance, ranging from 3:l^13^ to 14:l with peak incidence occurring in the third through fifth decades.^14^, ^15^, ^16^ SSc is more frequent and severe in African‐Americans.^3^, ^17^, ^18^

Dental and facial manifestations include: trismus (reduced interincisal distance)^19^; secondary microstomia (reduced interlabial commissure distance)^20^, ^21^, ^22^; dental caries^23^, ^24^, ^25^, ^26^; mask‐like appearance; muscular atrophy; thin lips; xerostomia^19^, ^25^, ^27^; rigidity with or without color change^28^ of tongue and lips; periodontal ligament widening^29^, ^30^, ^31^, ^32^, ^33^, ^34^; periodontal attachment loss^26^, ^35^; trigeminal neuralgia^36^, ^37^, ^38^; telangiectasia^19^, ^39^; oral mucosal/gingival fibrosis^2^, ^40^; gingival recession and stripping of attached gingiva^26^, ^41^; gastroesophageal reflux disease^42^; temporomandibular disorders^43^, ^44^ and resorption of the angle of the mandible^45^, ^46^, ^47^, ^48^; as well as the coronoid process and the condyle.^49^, ^50^, ^51^ The resorption has, on occasion, been so severe as to cause pathologic fracture of the mandible.^52^ These condylar changes may result in the temporomandibular joint symptoms of clicking, popping, and crepitus.^43^, ^44^ (See Table 1: Oral & Facial Manifestations of Scleroderma).

**Table 1:** Oral & Facial Manifestations of Scleroderma

Characteristic	Incidence	Reference(s)
Trismus	80%‐90%	Said et al,^19^ Jung et al,^26^ Baron et al,^22^ Bajraktari et al^21^
Microstomia	80%‐90%	DuBrul et al,^20^ Bajraktari et al,^21^ Baron et al^22^
Mask‐like cutaneous appearance	80%‐90%	Said et al,^19^ Jung et al^26^
Dental caries	3× Controls^a^	Wood et al,^23^ Baron et al,^22^ Albilia et al,^42^ Dagenais et al^50^
Temporomandibular disorders	80%+	Ferreira et al,^43^ Crincoli et al^44^
Telangiectasia	70%	Nagy et al,^39^ Said et al,^19^ Bajraktari et al^21^
Periodontal ligament widening	30%‐66%	Marmary et al,^29^ Auluck et al,^30^ Krogh et al,^31^ Anbiaee et al,^32^ Jung et al,^26^ Said et al,^19^ Rowell et al^34^
Gingival recession and stripping	2× Controls^a^	Eversole et al,^41^ Jung et al^26^
Periodontal attachment loss	2x Controls^a^	Pischon et al,^35^ Jung et al,^26^ Siefert et al,^33^
Xerostomia	25%‐71.2%	Said et al,^19^ Jung et al,^26^ Nagy et al,^39^ Chu et al,^25^ Vincent et al,^27^ Bajraktari et al^21^
Mandibular resorption	8.6%‐50%	Auluck et al,^30^ Rubin et al,^45^ Doucet et al,^46^ Pogrel et al,^49^ Dagenais et al,^50^ Jagger et al,^51^ Mugino et al,^52^ Siefert et al,^33^ Haers et al^48^
Trigeminal neuralgia	3%‐17%	Mohyuddin et al,^36^ Farrel et al,^37^ Jung et al,^26^ Amaral et al,^38^ Vincent et al,^27^ Bajraktari et al^21^

a Scleroderma patients’ incidence was two or three times control group incidence.

## 2 DESCRIPTION OF THE CASES

### 2.1 Case 1

A 34‐year‐old Caucasian female diagnosed in 2006 with scleroderma (systemic sclerosis) presented to The Ohio State University General Practice Residency clinic for comprehensive dental care with limited opening of her jaws. Physical examination revealed a cooperative, well‐developed, well‐nourished female with stiff posture, generalized reduced mobility and visible increase in facial skin thickness and rigidity. Saliva flow was slightly diminished. Initial interincisal distance (ICD) was 22 mm. Inter commissure measurement (ICM) was 40 mm with upper cuspid to cuspid distance of 48 mm. (See Table 2: Inter incisal & Intercommissure Distances in Two Scleroderma Patients).

**Table 2:** Interincisal & intercommissure distances in two scleroderma patients

Patientcase #	Pretreatment interincisal distance (ICD)	Pretreatment intercommissure measurement (ICM)	Treatment protocol	Posttreatment interincisal distance (ICD)	Posttreatment intercommissure measurement (ICM)	Percent change ICD	Percent change ICM
Case 1	22 mm	40 mm	PTX & Vitamin E	25 mm	42 mm	+13.6%	+5%
Case 2	26 mm	42 mm	PTX & Vitamin E + TheraBite	30 mm	46 mm	+15.4%	+9.5%

Abbreviation: PTX, Pentoxifylline.

Past medical history included: food poisoning; gastroesophageal reflux; generalized muscular weakness; temporomandibular disorder; pneumonia; and other upper respiratory infections. Previous uneventful surgeries were as follows: Cesarean Section 2005; minor foot surgery 2006; multiple port placements and removal of upper and lower second and third molar teeth in 2010. Social and family history were noncontributory. Regular medications were as follows: dexlansoprazole; diclofenac sodium 1% gel; potassium chloride; ranitidine hcl; norgestimate‐ethinyl estradiol; and tizanidine.

Panoramic radiograph and cone beam computed tomography revealed internal plus external resorption of numerous anterior and posterior teeth. Bilateral coronoid process resorption was also discovered. Generalized widening of posterior tooth periodontal ligaments was not evident. Bite wing radiographs did show eight existing restorations and eight new interproximal carious lesions with moderate penetration into dentin.

Periodontal examination indicated significant attachment loss on the buccal surface of the upper right second premolar.

Planned treatment consisted of: multiple amalgam, glass ionomer, and composite resin restorations; endodontic therapy for the upper right central incisor and lower right first bicuspid followed by a crown for the lower right first bicuspid. All procedures were to be performed under local anesthesia. An occlusal guard was provided.

Pentoxifylline 400 mg three times a day and Vitamin E 400 IU twice a day were prescribed. When this patient returned for dental treatment 1 month later, her ICD had increased to 25 mm (+13.6%). ICM also increased to 42 mm (+5%). (See Table 2: Interincisal & Intercommissure Distances in Two Scleroderma Patients).

### 2.2 Case 2

A 54‐year‐old African American female diagnosed with scleroderma (systemic sclerosis) also presented to The Ohio State University General Practice Residency clinic for comprehensive dental care with limited opening of her jaws. Physical examination revealed a cooperative, well‐developed, well‐nourished female with unlabored respirations, stiff posture, flexion contractures, generalized reduced mobility and slightly visible increase in facial skin thickness and rigidity. Saliva flow seemed adequate. Initial interincisal distance (ICD) was 26 mm. Inter commissure measurement (ICM) was 42 mm with upper cuspid to cuspid distance of 50 mm. (See Table 2: Interincisal & Intercommissure Distances in Two Scleroderma Patients).

Past medical history included sclerodermal interstitial lung disease and sickle cell trait. Previous uneventful surgeries were Cesarean sections ×2 and tubal ligation. Current medications were albuterol, ferrous sulfate, mycophenolate mofetil, potassium chloride, fluconazole, albuterol sulfate, and 1.1% sodium fluoride gel.

Panoramic radiograph revealed resorption of both angles of the mandible. Bitewings showed eight existing composite restorations and four grossly carious teeth.

Periodontal examination showed multiple teeth with attachment loss.

Dental treatment plan was to remove the upper right second and third plus lower left third molars along with the lower left first bicuspid and to restore the lower right third molar with composite using local anesthesia**.** An occlusal guard was provided.

Pentoxifylline 400 mg three times a day and vitamin E 400 IU twice a day were prescribed. A TheraBite^®^ device was given to the patient. The patient was instructed to exercise four times a day performing 6‐8 repetitions held for 10‐15 seconds each. When this patient returned for dental treatment one month later, her ICD had increased 4 mm (+15.4%). ICM also increased 4 mm (+9.5%). (See Table 2: Interincisal & Intercommissure Distances in Two Scleroderma Patients).

This patient reduced her pentoxifylline dosage from three times a day to two times a day.

## 3 DISCUSSION AND BRIEF REVIEW OF LITERATURE

Dental management of scleroderma focuses on trismus and microstomia. One large study concluded: “physicians may be disregarding issues related to oral health.”^53^

Previous therapies have included: thumb in cheek plus interocclusal tongue blade isometrics^54^; mouth stretching and facial massage^55^; facial grimacing exercises^56^; and carbon dioxide laser treatments.^57^ Drug treatments include: colchicine^58^; Gleevec^™^ 59, 60; D‐penicillamine,^61^, ^62^; chlorambucil^63^, ^64^;cyclophosphamide^65^; angiotensin converting enzyme inhibitors^66^; calcium channel blockers^66^; and pentoxifylline plus vitamin E.^1^, ^66^ Literature review suggests that pentoxifylline plus vitamin E is the safest, best tolerated, most effective pharmacologic treatment for dental scleroderma patients.^1^, ^58^, ^59^, ^60^, ^61^, ^62^, ^63^, ^64^, ^65^, ^66^, ^67^, ^68^, ^69^, ^70^, ^71^, ^72^, ^73^, ^74^, ^75^ (See Table 3: Summary of Cited Publications Documenting Use of Pentoxifylline and/or Vitamin C and Reported Complications).

**Table 3:** Summary of Cited Publications Documenting Dosage of Pentoxifylline and/or Vitamin C and Reported Complications

First author of publication	Daily dosage of pentoxifylline	Daily dosage of vitamin E	Reported complications
deSouza et al^1^	800 mg	800 Units	None
Delanian et al^69^	800 mg	1000 Units	None
Herrick et al^72^		270 Units	None
Ostojic et al^73^		400 Units	None
Tulsyan et al^68^	Various		None
Kamimura et al^74^		400 mg	None

Pentoxifylline, a theobromine methylxanthine analog, is frequently prescribed on label for peripheral arterial disease and claudication. Pentoxifylline works mainly because of its capacity to deform hemaceas and for its vasodilatation effect.^67^, ^68^ This drug can also decrease levels of tumoral necrosis factor (TNF), an inflammatory cytokine responsible for increased endothelial expression of adhesion molecules and for IL‐6 production^69^, ^70^ Pentoxifylline decreases fibroblast collagen and extracellular matrix production and reduces dermal fibroblast proliferation while increasing collagenase activity.^70^ In SSc, pentoxifylline has been described as a possible antifibrotic drug^71^ that may reduce vascular damage.^70^

Vitamin E has been used to treat SSc.^72^, ^73^, ^74^, ^75^ Vitamin E seems to have an antioxidant effect increasing free radicals excretion and also stabilizing cellular and lysosomal membranes.^73^ This could improve circulation and reduce tissue damage in scleroderma patients.

Pentoxifylline and vitamin E have diverse features altering the vascular component and/or the fibrotic process of SSc. This drug combination might be effective for dental patients with trismus, microstomia, and other disease‐related conditions.

**Figure 1:**
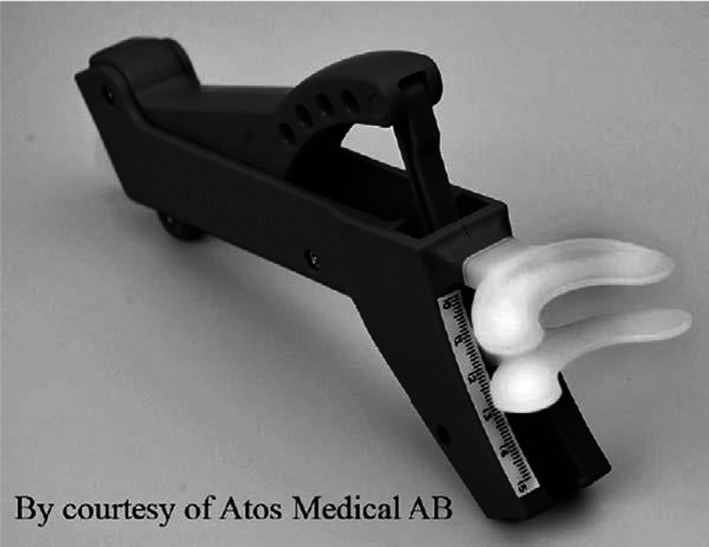
TheraBite® Device

TheraBite Jaw Motion Rehabilitation System^™^ is a portable device specifically designed to treat trismus. TheraBite Jaw Motion System^™^ provides stretching and passive motion for jaw rehabilitation therapy. One study achieved 5 mm increases in interincisal distances in a month using TheraBite^®^ to treat trismus.^76^(See Figure 1: Thera Bite^®^ Device).

Internal and external resorption of numerous teeth observed in case 1 is an uncommon finding in SSc.

## 4 CONCLUSIONS

Pentoxifylline plus vitamin E with TheraBite^®^ seems to be effective reducing trismus in scleroderma patients. Additional clinical research is needed.

## ETHICAL APPROVAL STATEMENT

Both patients signed informed consent forms including record use for publication.

Literature review searched PubMed for keywords: Scleroderma, Trismus, Systemic Sclerosis, SSc, TheraBite®, Pentoxifylline, and Vitamin E.

## CONFLICT OF INTEREST

There were no conflicts of interest or external funding sources to disclose.

## AUTHOR CONTRIBUTIONS

All authors reviewed the manuscript and submitted edits to the manuscript. Each author cited, read, and verified 5‐8 references.
